# Doxorubicin-Based Hybrid Compounds as Potential Anticancer Agents: A Review

**DOI:** 10.3390/molecules27144478

**Published:** 2022-07-13

**Authors:** Sijongesonke Peter, Sibusiso Alven, Rejoice Bethusile Maseko, Blessing Atim Aderibigbe

**Affiliations:** 1 Department of Chemistry, University of Fort Hare, Alice 5700, South Africa; 201414787@ufh.ac.za (S.P.); 201214199@ufh.ac.za (S.A.); 2 Department of Chemistry, School of Science and Technology, Sefako Makgatho Health Sciences University, Ga-Rankuwa 0208, South Africa

**Keywords:** cancer, doxorubicin, hybrid compounds, drug resistance, drug toxicity, chemotherapeutic drugs

## Abstract

The scarcity of novel and effective therapeutics for the treatment of cancer is a pressing and alarming issue that needs to be prioritized. The number of cancer cases and deaths are increasing at a rapid rate worldwide. Doxorubicin, an anticancer agent, is currently used to treat several types of cancer. It disrupts myriad processes such as histone eviction, ceramide overproduction, DNA-adduct formation, reactive oxygen species generation, Ca^2+^, and iron hemostasis regulation. However, its use is limited by factors such as drug resistance, toxicity, and congestive heart failure reported in some patients. The combination of doxorubicin with other chemotherapeutic agents has been reported as an effective treatment option for cancer with few side effects. Thus, the hybridization of doxorubicin and other chemotherapeutic drugs is regarded as a promising approach that can lead to effective anticancer agents. This review gives an update on hybrid compounds containing the scaffolds of doxorubicin and its derivatives with potent chemotherapeutic effects.

## 1. Introduction

Cancer is one of the life-threatening diseases worldwide [1]. The lack of awareness and effective chemotherapeutic agents has contributed to an increase in cases of cancer worldwide. Poor dietary habits, ageing, environmental factors, and lifestyle behaviours also contribute to an increase in the number of cancer cases [2,3]. Presently, more than 19 million cases and 10 million deaths were reported in 2020, and by 2040, it is predicted that the number could increase rapidly with more people requiring chemotherapy. There are more than 30 cancer types in the world including lung, breast, colon, stomach, prostate, liver, and brain cancer, with breast, lung, and colorectal cancer being the most reported cases [4]. Additionally, 90–95% of them are caused by gene mutations with 5–10% caused by inherited genetics [5]. Some reports have indicated that Africa and Asia are two continents with more deaths caused by cancer than the developed countries [3,4,5,6].

**Figure 1:**
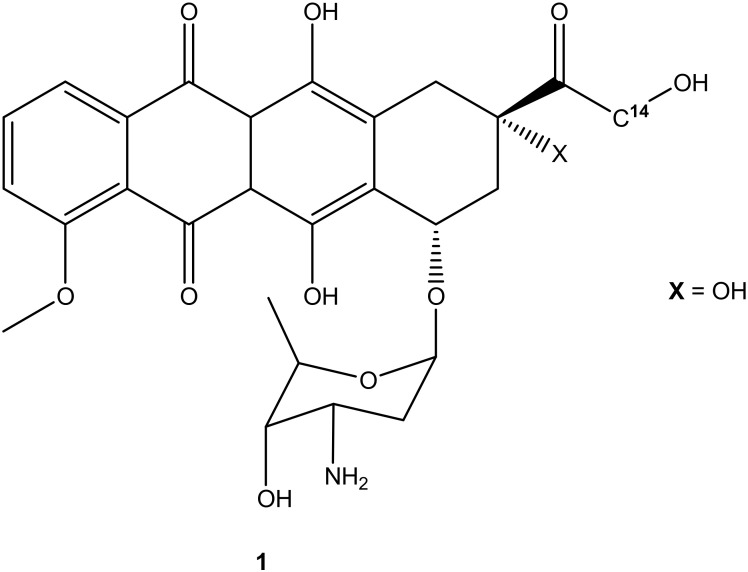
Chemical structure of doxorubicin (**1**).

The search for effective treatments for different cancers is a pressing need and is ongoing. Many antitumor compounds have been reported to exhibit promising effects. Doxorubicin (Figure 1), also known as adriamycin, is one of the derivatives that was extracted from *Streptomyces peucetius* in the late 1960s after pirarubicin, daunorubicin, epirubicin, idarubicin, and aclarubicin (anthracycline derivatives) [7,8]. It is regarded as one of the most used anticancer compounds from its class (anthracycline derivatives), together with epirubicin because of the antitumor effect against most cancers such as breast, lung, leukemia, brain, and lymphoma. Other compounds from the anthracycline class such as idarubicin and daunorubicin are mostly active against lymphoma and leukemia [8]. Doxorubicin and its derivatives are effective on most tumor cells. However, its high therapeutic effects and clinical applications are compromised due to its high hydrophilic nature, short half-life, low bioavailability, and high volume of distribution, which require high doses for effective treatment, resulting in side effects such as cardiotoxicity, extravasation, nephrotoxicity, and myelosuppression [9,10]. Furthermore, its efficacy is compromised due to drug resistance [11]. Although doxorubicin is regarded as an effective anticancer drug, its use has its pros and cons, resulting in the need to develop potent doxorubicin hybrid compounds that can overcome drug resistance with reduced side effects. Thus, this review reports the efficacy of doxorubicin derivatives in vitro and in vivo, an update on the development of doxorubicin hybrid compounds, the side effects associated with the use of doxorubicin for the treatment of cancer, active sites on doxorubicin suitable for modification, and the possible solutions to improve the anticancer effects of doxorubicin.

## 2. Side Effects Associated with Doxorubicin

Doxorubicin is used as the first-line treatment for myriad carcinomas. However, its undesired side effects such as cardiotoxicity, gonadotoxicity, and renal toxicity are compromising its chemotherapeutic effect. Its poor distribution, lack of solubility, and nonspecific action are some limitations associated with doxorubicin [12,13]. Additionally, most of the lethal side effects of doxorubicin are developed in a dose-dependent mode. Its antitumor effectiveness is mostly when administered in high doses [12,13]. High doses of doxorubicin can cause gonadotoxicity, causing infertility in some patients due to testicular toxicity (resulting in reduced testicular weight, lowered testosterone level in circulation, and reduced testicular lipids, affecting the sperm motility and zinc deficiency in the testicle) and ovarian toxicity, causing a reduced size of the ovary [12,13,14,15,16,17,18]. Moreover, high doses of doxorubicin can cause irreversible cardiotoxicity and are not used to treat patients with poor heart function, especially elderly patients [12,13,14,15,16,17,18].

Apart from dose-dependent side effects, brain, liver, and kidney toxicity are also caused by the poor transportation of doxorubicin into the target tumor [19,20,21]. The mechanism of action of doxorubicin in the treatment of brain tumors results in some patients experiencing cognitive impairments. However, these cognitive impairments are mild and can be reversed after one-year post-treatment [2,19,20,21,22,23,24]. A high concentration of doxorubicin can cause liver damage by producing a high concentration of reactive oxygen species (ROS) during the metabolism, and excess ROS can result in DNA damage. It also reduces the inorganic phosphates in the liver, leading to pathological conditions in the hepatocytes [2,19,20,21,22,23,24]. Additionally, doxorubicin causes damage to the glomerular podocytes, causing proteinuria and nephropathy, processes that cause toxicity to the kidney. Disruption of the mitochondria’s normal functioning by doxorubicin can result in renal failure. Urticaria, fever, cutaneous injuries, vomiting, necrosis, and ulceration are other toxic effects associated with doxorubicin, which can be fatal in some cases [2,13,24]. 

### 2.1. Doxorubicin Mechanism of Action

Many drugs interact with DNA and disrupt the processes of cell replication, resulting in enhanced anticancer activity. The quinone moiety and amino group on doxorubicin contribute to its anticancer activity [8,25]. Doxorubicin’s mode of action is still not well understood. However, there are certain processes such as topoisomerase II inhibition, the release of cytochrome c from mitochondria, intercalation into DNA, DNA unwinding/separation, increase in alkylation, and the generation of free radicals leading to oxidative stress that are associated with doxorubicin’s cytotoxic effects [11]. All of the aforementioned processes are associated with doxorubicin action but the disruption/poisoning of enzyme topoisomerase II progress by inhibiting the macromolecular biosynthesis through intercalation with DNA using sugar moieties and the cyclohexane ring of doxorubicin, resulting in DNA damage (cell death), is still the main mode of action of doxorubicin. Some studies have shown that doxorubicin disrupts the mitochondrial and nuclear DNA through binding, resulting in tumor cell death [25,26,27]. Topoisomerase II is responsible for the survival and division of the cancer cell; hence, its poisoning leading to DNA damage is vital as cancer cells are more vulnerable to DNA breaks than healthy cells [8,13]. Another mode of action of doxorubicin is its ability to produce excess Reactive Oxygen Species (ROS) such as hydrogen peroxide (H_2_O_2_) and superoxide anions (O_2_^−^), which are converted into free radicals (OH), resulting in cell membrane lipids, protein, and DNA damage that can lead to oxidative stress, resulting in tumor cell death via apoptosis [8,13].

### 2.2. Doxorubicin Mechanism of Resistance

The mechanism of doxorubicin resistance is important because it highlights drug targets for the development of novel and effective doxorubicin derivatives with limited shortcomings [28]. Doxorubicin drug resistance is not well understood, but it is caused by several factors such as the uniqueness of tumor properties, disruption of the apoptosis process, inhibition of autophagy, alterations to the expression of the topoisomerase II, gene mutation, and drug transport [29,30]. Generally, topoisomerase II is responsible for doxorubicin resistance because doxorubicin attaches to the alpha-isoform of Topoisomerase II for effective apoptosis; thus, its resistance comes when there is a reduction in α-isoform of topoisomerase II, resulting in doxorubicin attaching to the β-isoform of topoisomerase, which is less sensitive to doxorubicin, leading to drug resistance. Furthermore, drug transport also causes doxorubicin resistance because it fails to reach and accumulate in the targeted tumor cells due to drug efflux. It is challenging for doxorubicin to pass the blood-brain barrier, resulting in its inability to reach the brain tumor cells [29,30]. Another factor triggering doxorubicin resistance is the disruption of p53 (responsible for controlling cell death and cell division) processes through mutations or deletion [29,30]. Therefore, the general mechanism for doxorubicin resistance is triggered because it fails to bind with mitochondrial and nuclear DNA due to the aforementioned factors (drug efflux), which result in DNA damage that can cause cell death. 

### 2.3. Possible Solution to Overcome Doxorubicin Negative Effects 

Doxorubicin is a potent anticancer drug; however, its side effects are a major drawback, revealing a pressing need to develop effective anticancer therapeutics. Different options have been utilized to overcome the negative side-effects of doxorubicin using drug delivery systems that include nanoparticles, liposomes, polymeric micelles, polymer-drug conjugates, ligand-based DOX-loaded nanoformulations, etc. [11,27]. Doxorubicin has also been used in combination therapy with other anticancer drugs to overcome multidrug resistance [11,27]. Furthermore, the modification of the doxorubicin structure is also among the significant solutions that can overcome doxorubicin’s side-effects reported by different researchers [11,27]. Doxorubicin consists of two active sites (–NH_2_ and –OH), which make it suitable for modification. Therefore, the hybridization of doxorubicin with other anticancer pharmacophores is reported to be a significant strategy to overcome doxorubicin’s side effects by different researchers [11,27]. The issue of drug toxicity is a major drawback in the treatment of cancer; the hybrid derivatives that have been investigated until now are reported in Section 3 of this review. 

## 3. Anticancer Activity of Doxorubicin and Its Derivatives

To overcome the limitations associated with doxorubicin such as short retention time, cardiotoxicity, multidrug resistance, and rapid excretion, efforts have been made to enhance its chemotherapeutic efficacy via chemical modifications to form doxorubicin derivatives. Some reports have proved that the alteration of the compounds’ pharmacokinetics through chemical modifications can result in effective anticancer drugs with reduced toxicity and limited side effects [27,31]. Thus, hybrid derivatives of doxorubicin containing cholesterol scaffolds, fatty acids, anti-oxidants, anti-estrogen, etc. are discussed as promising anticancer agents. They have been reported to exhibit improved efficacy in terms of overcoming cardiotoxicity, a common side-effect of doxorubicin and reducing the risk of development of drug resistance. 

### 3.1. Cholesteryl-Doxorubicin Derivatives

**Figure 2:**
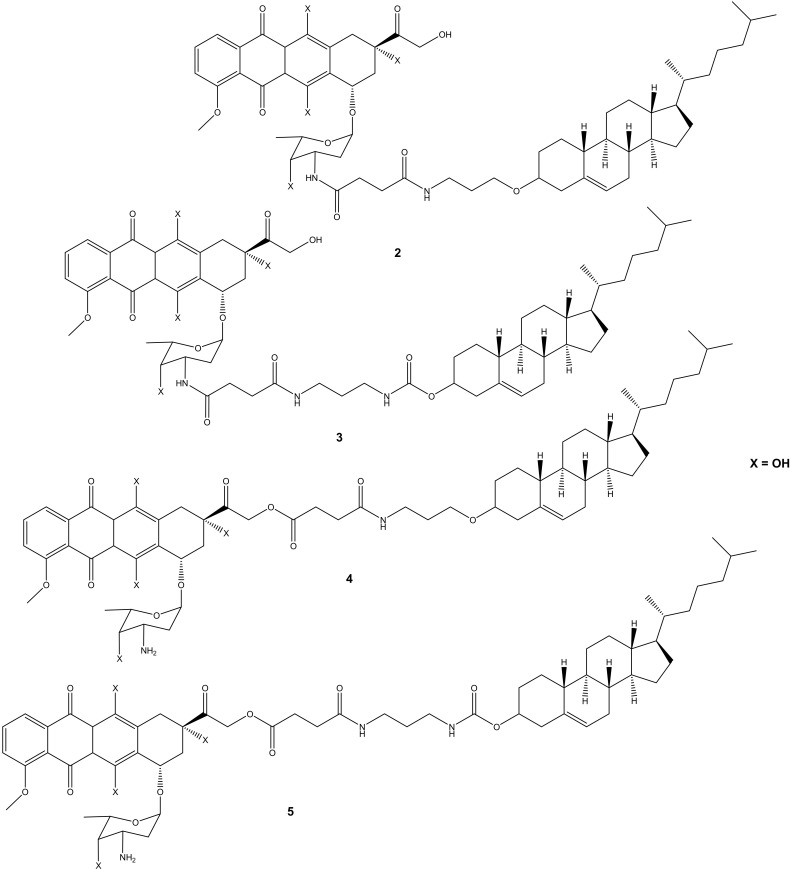
Chemical structures of cholesteryl-doxorubicin compounds synthesized through amidation and esterification (**2**–**5**).

Choi et al. synthesized cholesteryl-doxorubicin compounds (Figure 2) by reacting doxorubicin with cholesterol via the hydroxyl at position C-14 and amino groups of doxorubicin to improve doxorubicin’s anticancer effect. The anticancer activity of these compounds was tested against four human cancer cell lines (i.e., cervical (HeLa), breast (MDA MB 231), lung (A549), and human breast (MCF7)) [31]. Two compounds (**2** and **3**) were synthesized through chemical modification using the amino group (-NH_2_), whereas compounds **4** and **5** were modified using the hydroxyl group (-OH). Moreover, during the synthesis of compounds **4** and **5** (through -OH group), the amino group was protected using 9-fluorenylmethyl succinimidyl carbonate because the amino group is reactive [31]. Compounds **2** and **3** exhibited poor anticancer activity against all cancer cell lines, whereas compounds **4** and **5** displayed an anticancer activity that was comparable to doxorubicin with half-maximal inhibitory concentration IC_50_ values in the range of 8.03–27.88 μM (Table 1). Compound **5** was more effective compared to compound **4** against all the cancer cell lines used in the study. Modifying the amino group affected the anticancer effect of these doxorubicin derivatives, revealing the importance of the amino-functional group on doxorubicin [31,32].

### 3.2. Doxorubicin-Fatty Acyl Derivatives

**Figure 3:**
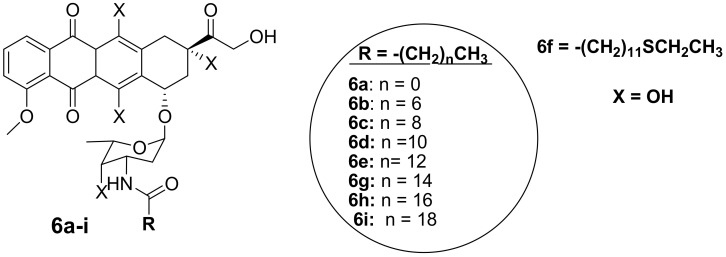
Chemical structures of doxorubicin-fatty acyl derivatives synthesized via the amino group (**6a**–**i**).

Polyunsaturated fatty acids are useful for human health and enhanced immune systems. Reports have revealed that fatty acids can improve the activity of anticancer drugs by extending the drug’s half-life and improving the drug’s bioavailability in vivo [33,34,35,36,37,38]. Chhikara et al. synthesized a series of doxorubicin-fatty acyl amide derivatives (**6a**–**i**) (Figure 3) through chemical modification of the amino group to improve the anticancer activity and lipophilicity of doxorubicin. The antiproliferation activity of the compounds against breast (MDA-MB-468), leukemia (CCRF-CEM), colon (HT-29), and ovarian (SK-OV-3) cancer cell lines was evaluated [9]. The synthesized compounds displayed enhanced lipophilicity compared to the parent drug, doxorubicin, and an increase in the fatty acyl moiety chain was responsible for the improved lipophilicity of the drug. Conversely, the introduction of the fatty acyl chains into doxorubicin via the amino group compromised the anticancer activity of these compounds compared to doxorubicin, confirming that the free amino group is vital for the anticancer activity of doxorubicin [9]. After 96 h of incubation at a concentration of 1 μM, **6d** was the most potent compound against SK-OV-3 and HT-29 cancer cells by 58% and 64%, respectively. Thus, these compounds were more active against colon and ovarian cancer than breast and human leukemia cancer cell lines [9]. The disruption of the amine group influenced the anticancer activity of these compounds and further studies need to be conducted on the modification of doxorubicin without compromising its therapeutic effect [9]. 

**Figure 4:**
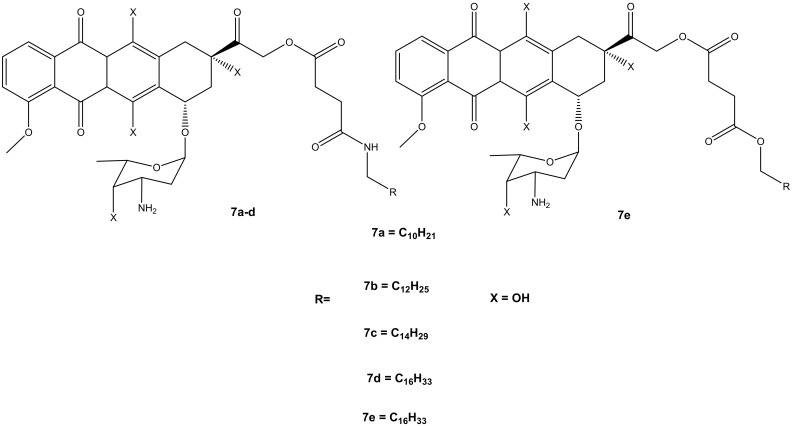
Chemical structures of the second generation of doxorubicin-fatty acyl derivatives synthesized via the hydroxyl group in position C-14 (**7a**–**e**).

Thus, the second generation of doxorubicin-fatty acyl amide derivatives (**7a**–**e**) (Figure 4) was synthesized and reported by Chhikara et al. During the synthesis, the amino group of a sugar moiety of doxorubicin was not disrupted; instead, the chemical modification was performed via the hydroxyl group aglycone moiety at position C-14 using succinic anhydride followed by the addition of tetradecanol or fatty amines [9,10]. These derivatives were evaluated in vitro for their anticancer activity against the same tumor cell lines, which were used for the first generation of compounds synthesized by Chhikara et al. [10]. It was displayed that the inhibition of cell proliferation was influenced by time, and the compounds’ inhibitory effect after the 96 h of incubation was enhanced. Moreover, these compounds showed inhibition of cell proliferation that was comparable to the first generation after 96–120 h of incubation at a concentration of 1 μM against most of the cancer cell lines. The compounds’ antiproliferative activity was cell-specific with most of the compounds exhibiting an antiproliferative effect in this order: SK-OV-3 (54–62%) < MDA-MB-468 (49–63%), MDA-MB-361 (66–71%) < CCRF-CEM (66–77%) and HT-29 (60–77%) [10]. However, amide derivative **7c** (70.2%) exhibited an antiproliferative activity that was comparable to doxorubicin (69.1%) against MDA-MB-468, and it was slightly better than that of ester derivative **7e** on all the tumor cell lines. Compound **7c** displayed a promising anticancer activity compared to doxorubicin with prolonged retention, high cellular distribution, and higher cellular uptake in SK-OV-3 cells [10]. The conjugation of doxorubicin in the 14th position did not compromise its antiproliferative effect [10]. 

**Figure 5:**
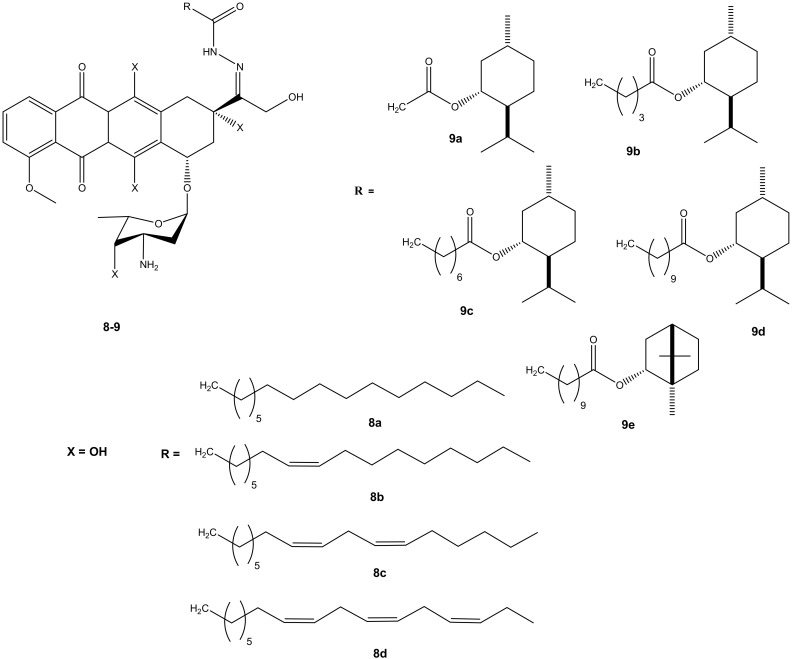
Chemical structures of doxorubicin *N*-acyl hydrazones derivatives (**8**–**9**).

Effenberger et al. conjugated doxorubicin with terpenyl and fatty acyl hydrazones to improve the cytotoxic effect of doxorubicin. A series of doxorubicin *N*-acyl hydrazone compounds were synthesized (**8** and **9**) (Figure 5). They were evaluated in vitro for their cytotoxic effects against four different human tumor cell lines (breast (MCF-7/Topo), leukemia (HL-60), cervix (KB-V1/Vbl) and melanoma (518A2) tumor cell lines). Most of the synthesized compounds exhibited improved anticancer activity when compared to their parental drug, doxorubicin. Their anticancer activity was influenced by the presence of selected substituents [39,40]. After 72 h of incubation, most of the synthesized doxorubicin acyl hydrazones compounds (**8a**–**d**) displayed anticancer activity against all of the cancer cells lines used in the study, except **8c.** Compound **8a** exhibited the greatest cytotoxic effect and **8c** exhibited the lowest. Compound **8a** (IC_50_: 1.02 ± 0.04) exhibited a better cytotoxic effect against the multidrug resistance cervix (KB-V1/Vbl) cancer cell line and it was three times more cytotoxic than doxorubicin on the cancer cell line [39,40]. Doxorubicin- terpenylhydrazones (**9a**–**e**) exhibited a comparable anticancer activity of doxorubicin against all the tumor cell lines. However, compound **9d** displayed a more cytotoxic effect than doxorubicin against cervix (KB-V1/Vbl) and melanoma (518A2) [39,40]. 

**Figure 6:**
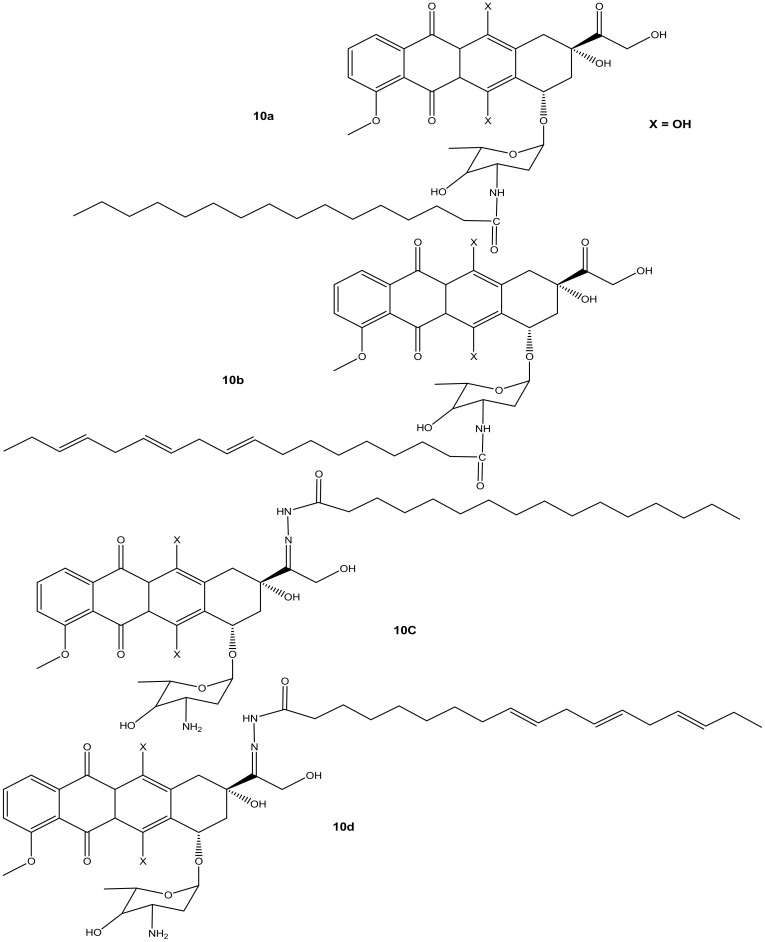
Chemical structures of doxorubicin-PA-α-LA derivatives conjugated via amide bond and hydrazone bond formation (**10a**–**d**).

Liang et al. synthesized doxorubicin derivatives (**10a**–**d**) (Figure 6) by conjugating doxorubicin with palmitic acid (PA) and α-linolenic acid (α-LA) via amide bond and hydrazone bond formation to attenuate doxorubicin’s toxicity toward normal cells and improve its chemotherapeutic effects. The cytotoxicity effects of these compounds were evaluated against three tumor cell lines (MCF-7, MDA-231, and HepG2) in vitro [41]. Among the synthesized compounds, only compound **10d** (with α-LA) exhibited a superior cytotoxic effect compared to doxorubicin against all the aforementioned cancer cell lines, as shown in Table 2. Modifying the amino group reduced the anticancer activity of compounds **10a** and **10c** compared to doxorubicin. Compound **10b** also displayed a reduced cytotoxic effect compared to doxorubicin, suggesting that the hydrazone bond formation did not improve the activity of compound **10b**. Compound **10d** exhibited reduced cytotoxicity on MDA-MB-231 cells, but the highest cytotoxicity was significant against HepG2 cells [41]. Due to the promising results, compound **10d** was evaluated in vivo and this compound displayed stronger tumor inhibition growth than doxorubicin. The compound stability in serum, high rate of doxorubicin release, and improved distribution rate of doxorubicin in the tumor tissues may have resulted in its enhanced anticancer activity, and there is a need for further studies to fully understand its mode of action [41].

**Figure 7:**
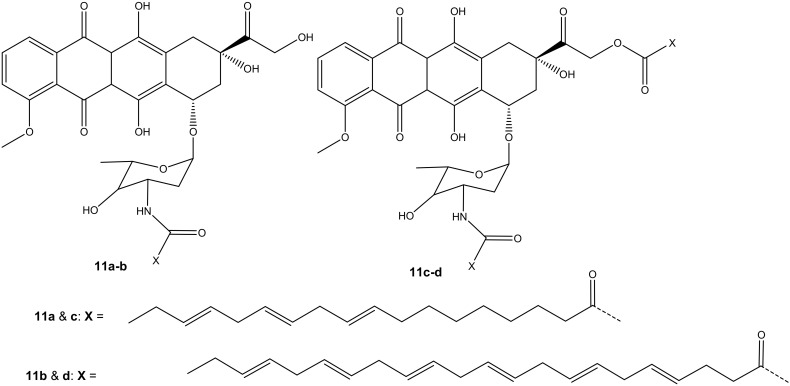
Chemical structures of doxorubicin conjugated with DHA and α-LA conjugated through amidation and esterification (**11a**–**d**).

Mielczarek-Puta et al. reported doxorubicin conjugated with docosahexaenoic (DHA) and α-LA (unsaturated fatty acids) and confirmed the expected structures of the compounds (**11a**–**d**) (Figure 7). The structures were studied using High-Resolution Mass Spectrometry (HRMS) and Nuclear Magnetic Resonance (^1^H NMR and^13^C NMR). Their cytotoxicity effect was investigated on three human tumor cell lines, SW480, SW620, and PC3, with Chinese hamster lung fibroblasts (V79) used as a control [42]. Compounds **11a**–**b** were synthesized via amide bond formation and compounds **11c**–**d** were double-substituted through both ester and amide linkages. Compounds **11c**–**d** prepared through amide and ester bond linkages exhibited a superior anticancer activity (i.e., higher cytotoxicity on cancer cell lines) to compounds **11a**–**b** that were formed through an amide bond. However, the compounds (**11a**–**d**) exhibited a lower anticancer activity than doxorubicin against all the tumor cells [42]. The IC_50_ values of **11c**–**d** conjugates were in the same range. However, the selective index (SI) factor showed that compound **11c** exhibited the highest SI factor when compared to other compounds [42]. 

### 3.3. Doxorubicin-Hydrazone Derivatives

**Figure 8:**
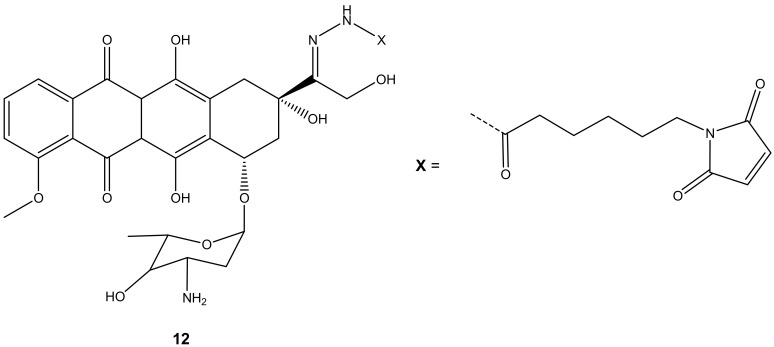
Chemical structure of doxorubicin-6-maleimidocaproyl hydrazone hybrid (**12**).

Graeser and co-workers synthesized doxorubicin-6-maleimidocaproyl hydrazone derivative, **12** (Figure 8), aiming to improve doxorubicin antitumor activity, and evaluated it against pancreas (ASPC-1), ovarian (A2780), lung (H209), and breast (3366) cancer xenograft models [43]. Compound **12** was effective against all the aforementioned cancer cells compared to doxorubicin, especially on lung (H209) cancer lines, as the results displayed that doxorubicin showed no effect on tumor volumes with an increasing factor of 16 compared to approximately 3.5 when treated with compound **12** after 44 days against lung cancer cell lines [43].

### 3.4. Doxorubicin Hybrid Containing Compounds with Antioxidant Activity

**Figure 9:**
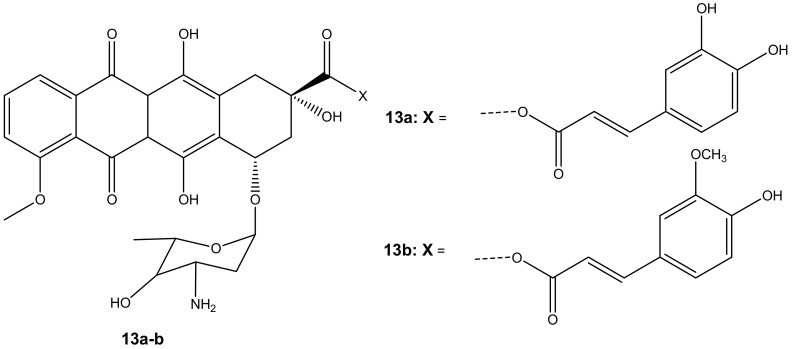
Chemical structures of doxorubicin-antioxidant derivatives synthesized via esterification (**13a-b**).

Chegaev et al. synthesized two compounds (**13a**–**b**) (Figure 9) to reduce the cardiotoxicity effect of doxorubicin caused by oxidative stress and to improve its therapeutic effects [44]. The compounds **13a**–**b** were synthesized by combining ferulic and caffeic acid with doxorubicin via esterification reactions (ester linkage) at position C-14. Two breast cell lines (MDA-MB-231 and MCF7) together with H9c2 cells (cardiomyocytes) were used to evaluate the cytotoxicity of these compounds in vitro [44]. The compounds displayed cytotoxicity comparable to the parent drug, doxorubicin, against both breast cancer cell models. The compounds also reduced oxidative stress [44]. However, **13b** was the most effective synthesized compound when compared to **13a**, and its cytotoxic effect was superior to doxorubicin on both breast cancer cells but less toxic on cardiomyocytes. The difference in lipophilicity or different rates of intracellular accumulation of the precursors of these compounds influenced their efficacy. The concentration of compound **13b** did not affect its cytotoxicity on resistant breast tumors. The compound displayed the same cytotoxic effect when administered at lower doses than doxorubicin, revealing its therapeutic efficacy even at a low concentration. The findings suggest that compound **13b** has the potential to reduce cardiac damage in patients with resistant breast cancer cells [44]. 

**Figure 10:**
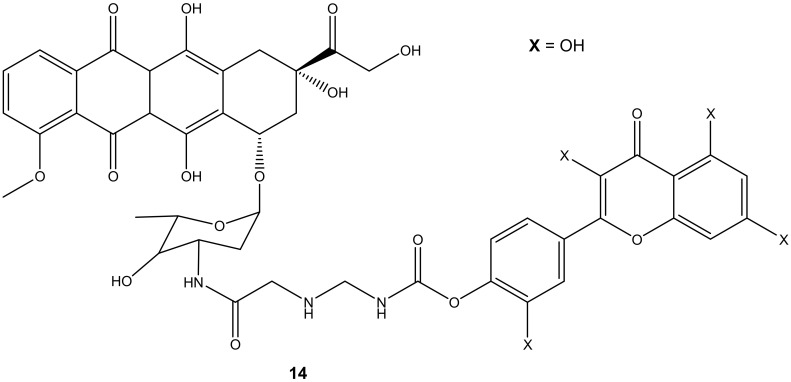
Chemical structure of quercetin-doxorubicin derivative synthesized via the amino group (**14**).

Doxorubicin derivative (**14**) (Figure 10) was synthesized by Alrushaid et al. by reacting doxorubicin with quercetin via the amino group of daunosamine moiety. The cytotoxicity of the compound was evaluated using triple-negative murine breast cancer cells in vitro [45]. Compound **14** was less toxic compared to doxorubicin or a mixture of doxorubicin and quercetin. The IC_50_ value further revealed that the compound’s cytotoxic effect was two-fold higher than the mixture of doxorubicin and quercetin which is attributed to the poor water solubility of the drug and slow release of the drug from the hybrid. The compound’s cardiotoxicity was also evaluated in rat and human cardiomyocytes, and it displayed a less toxic effect than doxorubicin on the cardiomyocytes cell lines used [45].

### 3.5. Formamidino-Doxorubicin Derivatives

**Figure 11:**
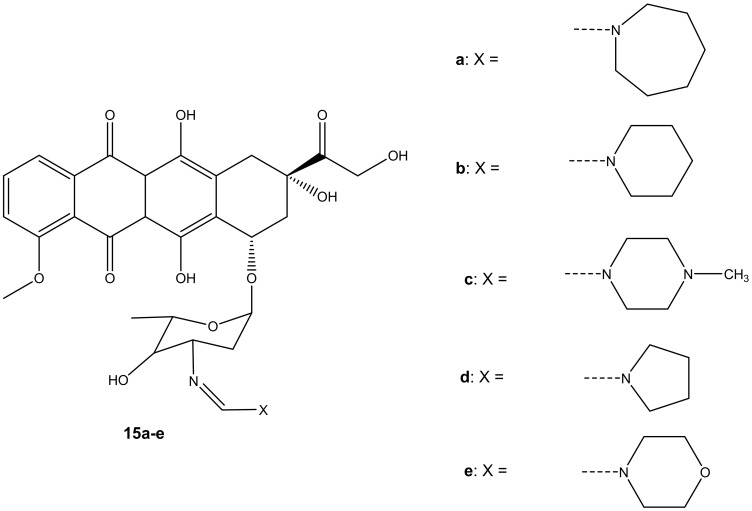
Chemical structures of formamidino-doxorubicin derivatives synthesized via the amino group (**15a**–**e**).

Five formamidino-doxorubicin derivatives, **15a**–**e** (Figure 11), were synthesized and reported by Marczaka et al. [46]. These compounds were prepared by substituting the amide group with a formamidine group with hexamethyleneimine, piperidine, *N*-methylpiperazine, pyrrolidine, and a morpholine ring on the daunosamine moiety. Their anticancer effects were studied using human ovarian cancer cells (SKOV-3). The derivatives were more cytotoxic than doxorubicin. Compounds containing a six-membered ring with heteroatoms such as **15c** and **15e** were more cytotoxic than those without heteroatoms such as **15a** and **15b** with IC_50_ values ranging between 82.42 ± 8.47 and 251.27 ± 19.3 nM (as shown on Table 3), suggesting that the presence of heteroatoms on the six-membered ring improved the anticancer activity of these compounds [46]. Furthermore, it was highlighted that substitution via the amino group compromised the anticancer activity of the doxorubicin derivatives. However, the presence of heteroatoms in the six-membered rings in these compounds improved the anticancer activity of these analogues [46]. In essence, the anticancer effect of doxorubicin derivatives might also depend on the dominant type of cell death induced by the compounds combined with doxorubicin. Moreover, developing novel anticancer agents that can induce apoptosis can be one promising approach to obtaining an effective anticancer therapy for the treatment of solid tumors [46,47,48,49,50,51]. 

### 3.6. Dexamethasone-Doxorubicin Derivative 

**Figure 12:**
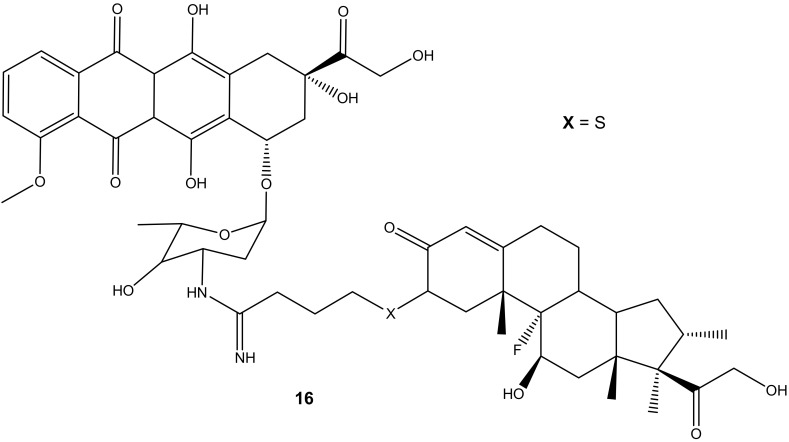
Chemical structure of dexamethasone-doxorubicin derivative synthesized via the amino group (**16**).

Dexamethasone-doxorubicin, (**16**) (Figure 12), was synthesized by Chaikomon et al. to improve doxorubicin’s antitumor activity and overcome multidrug resistance [52]. The compound was evaluated for apoptosis activity against MCF-7 in vitro [52]. The efflux of doxorubicin out of the tumor can result in drug resistance. The derivative was less cytotoxic than the parent drug, doxorubicin, with an IC_50_ value of 90.31 ± 7.3 µg/mL when compared to 2.8 ± 0.9 µg/mL of doxorubicin against MCF-7 cell lines. However, its ability to induce apoptosis without its uptake into the nucleus, improve cytosolic oxidative stress, and not be involved in the cell cycle makes it a promising anticancer agent. Its ability to generate ROS contributed to it overcoming the P-gp efflux pump that is responsible for multi-drug resistance. The overexpression of the P-gp efflux pump did not have any significant effect on the drug cytotoxicity [52].

### 3.7. Doxorubicin Derivative Containing Arimetamycin Scaffolds

**Figure 13:**
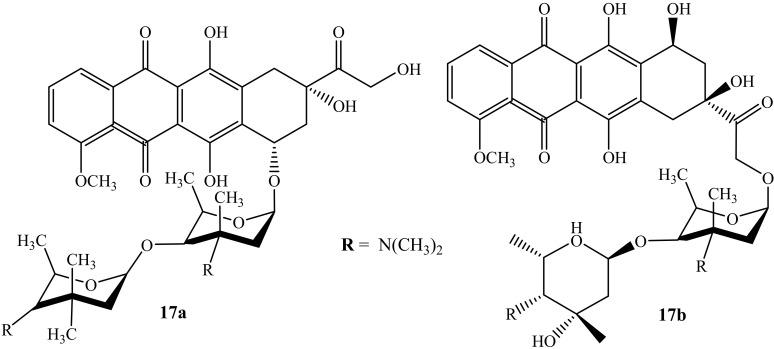
Chemical structure of doxorubicin modified by saccharides (**17a**–**b**).

Huseman et al. modified anthracycline hybrid compounds and evaluated their cytotoxicity activity against three cancer cell lines, i.e., colon (HCT116), breast (MDA-MB 231), and multi-drug-resistant lung cancer line (H69AR), to improve and overcome anthracycline’s resistance, in vitro [53]. Furthermore, H69AR is responsible for anthracycline resistance due to its overexpression of the multidrug resistance protein 1 (MRP1) efflux pump [53]. Among the synthesized compounds were two doxorubicin-containing hybrids (**17a** and **17b**) (Figure 13). Compound **17a** was the most potent compound when compared against its parent drugs, Arimetamycin A and doxorubicin, against all three cancer cell lines with TC_50_ values shown in Table 4 [53]. The site of the substitution influenced the cytotoxicity of the compound, as the modification of compound **17b** is on C14. Moreover, compound **17a**’s mechanism of action is still unclear; however, these results suggest that the modification of anthracyclines can be a promising approach for the treatment of cancer. 

**Figure 14:**
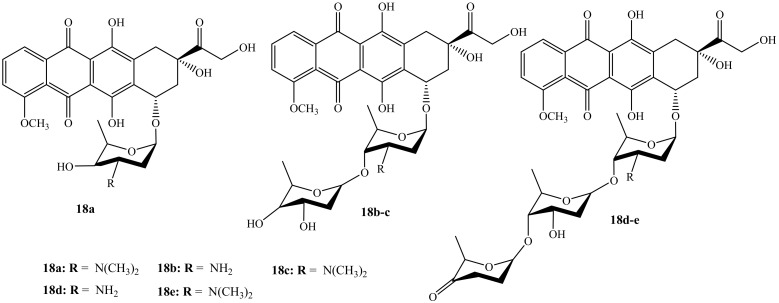
Chemical structures of the second generation of doxorubicin modified by saccharides (**18a**–**e**).

Wander et al. reported the synthesis (monosaccharides, disaccharides, and disaccharides) of doxorubicin hybrid compounds (**18a**–**e**) (Figure 14) through anthracycline’s structural modification using Yu’s gold-catalyzed glycosylation and IDCP-mediated glycosylations to improve the anticancer activity of the anthracyclines [54]. The anticancer activity of these compounds was tested on several human cancer cell lines. Furthermore, TopoIIα’s relocalization capacity, cellular uptake, histone eviction assays, and ability to damage DNA were also evaluated. Moreover, among the doxorubicin hybrids synthesized, compounds **18a** and **18e** exhibited improved cytotoxicity than their parent drug with other synthesized compounds’ cytotoxicity depending on the drug concentration. Additionally, *N,N*-dimethylation in the sugar moiety is suggested to be responsible for the improved cytotoxicity as methylated hybrids outperformed nonmethylated counterparts [54]. Compound **18e** was the most potent compound in the series and it was approximately 13 times more cytotoxic than doxorubicin in leukemia cell line (K562). 

### 3.8. Photoresponsive-Doxorubicin Hybrid

**Figure 15:**
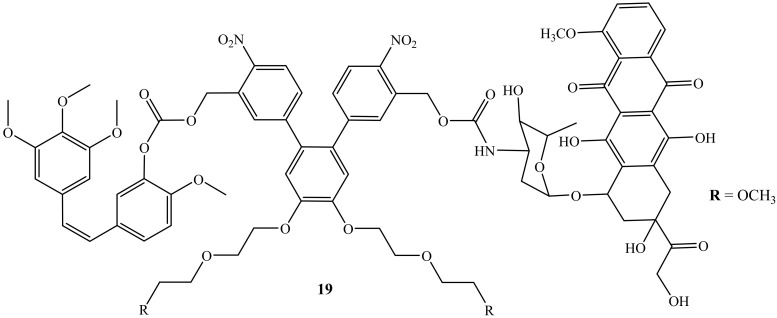
Chemical structure of doxorubicin combined with combretastatin A4 via the amino group (**19**).

Liu et al. reported a photoresponsive prodrug, **19**, which is a combination of doxorubicin and combretastatin A4 (Figure 15). The cytotoxicity of the prodrug was evaluated on human breast cancer cell lines (MDA-MB-231) [55]. Furthermore, photo-removable protecting groups (PPGs) are known as the drug transporter for the drug molecules and are reported as effective in the treatment of cancer as they bypass the challenges such as the potential long-term toxicity of the polymers or nanomaterials and biocompatibility [55]. However, this prodrug, **19**, displayed high efficacy when used in combination therapy, as the cell viability of this compound displayed improved cytotoxicity when compared to the parent drug, suggesting that the synergistic effect was achieved. Thus, this prodrug was reported as a promising drug combination against neovasculature and it is available for further biological application [55]. 

### 3.9. Steroidal Anti-Estrogen−Doxorubicin Bioconjugate

**Figure 16:**
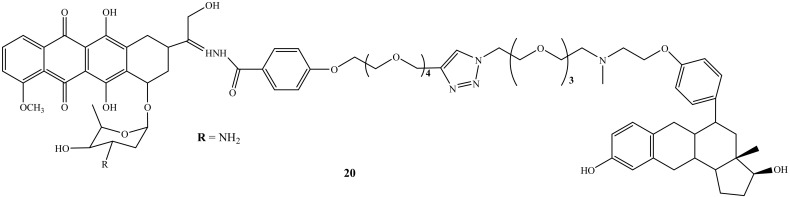
Chemical structure of steroidal anti-estrogen−doxorubicin bioconjugate (**20**).

Nonspecific action is one of the side-effects caused by chemotherapeutic agents, and as a result, the development of specific therapeutic agents is a pressing need to overcome this shortcoming. Thus, steroidal anti-estrogen−doxorubicin bioconjugate (**20**) (Figure 16) was synthesized by Dao et al. to reduce doxorubicin’s side effects by improving drug specificity and enhancing the doxorubicin efficacy. Moreover, the cytotoxicity and selective uptake of this biconjugate were evaluated against two human breast cancer cells lines, ER(−)-MDA-MB-231 and ER(+)-MCF-7 [56]. Compound **20** was approximately 70-fold more potent than its parental drug in promoting cell death and inhibiting cell proliferation. The presence of the anti-estrogenic component was significant for improved cytotoxicity and selectivity. In addition, several structural factors were noted in this study as they affect the activity of the hybrid compounds for breast cancer cells. The targeting group, type of linker, and binding site of estrogen receptors and anti-estrogen were noted as important structural factors affecting the efficacy of doxorubicin bioconjugate. Thus, Dao et al. suggested that the linker length must be increased to allow the ER-binding component to interact with the target protein while maintaining a stable bond with the parent drug [56].

### 3.10. Doxorubicin Computational Work

There is growing interest in the use of computational models as one of the analysis techniques in the development of doxorubicin derivatives. Some scientists used in silico computational modelling for new hybrids to predict their pharmacokinetics (binding affinity, cytotoxicity, modes of action, etc.) on tumor cell lines as well as normal cells [57,58,59,60,61,62]. Furthermore, molecular dynamic simulation is one of the powerful computational tools used to predict the information about interactions and physicochemical mechanisms of the drug [57]. This method has been used to study doxorubicin loaded in nanomaterials (e.g., nanocarriers and nanoparticles) by different researchers [57,58,59,60,61,62]. However, there is no published information on the use of this method for hybrid organic molecules containing doxorubicin scaffolds. Thus, the use of in silico pharmacokinetics prediction must be introduced when developing doxorubicin-based hybrid compounds with anticancer activity.

## 4. Future Perspectives and Conclusions

Cancer cases and deaths are increasing rapidly and the currently used chemotherapeutic agents suffer from some drawbacks. The development of new and effective chemotherapeutic drugs is a pressing need. Doxorubicin is one effective anticancer drug used to treat several types of cancer. However, its use in high concentrations leads to some side effects such as cardiotoxicity, renal toxicity, and gonadotoxicity. Doxorubicin is effective but dose-dependent, resulting in its adverse side effects. However, the chemical modification of doxorubicin with other drugs has been reported. The presence of functional groups such as hydroxyl and amino groups on doxorubicin makes it easy to modify. The amino group is sensitive and responsible for its anti-cancer activity and, therefore, it is important to avoid chemical modifications through the amino groups in future drug designs and developments. Thus, the position of the substitution must be considered carefully when developing new doxorubicin derivatives. Furthermore, there are limited data on doxorubicin derivatives containing scaffolds of other known anticancer drugs. This finding suggests that there is still a need for the development of more doxorubicin derivatives. The design of doxorubicin derivatives will limit the use of high doses of doxorubicin and result in effective drugs with less toxicity and dual targets. Generally, the fight against cancer needs several strategies; thus, the development of potent chemotherapeutic agents is a pressing need. 

## Figures and Tables

**Table 1:** IC_50_ values of compounds **4** and **5** against four cancer cell lines.

IC_50_ (μM)	Compound 4	Doxorubicin	Compound 5
MCF 7	9.94	4.39	9.69
A549	11.22	11.62	8.03
MDA MB 231	21.60	14.66	16.35
HeLa	27.88	7.18	8.03

**Table 2:** IC_50_ values of compound **10d** against three cancer cell lines.

IC_50_(μM)	Doxorubicin	Compound 10d
MDA-MB-231	4.3 ± 1.1	2.2 ± 0.7
MCF-7	3.6 ± 1.3	1.7 ± 0.3
HepG2	3.2 ± 0.7	1.3 ± 0.4

**Table 3:** IC_50_ values of compounds **15a-e** against SKOV-3 cancer cell lines.

IC_50_	doxorubicin	15a	15b	15c	15d	15e
SKOV-3	352.79 ± 25.04	251.27 ± 19.3	112.30 ± 9.55	82.42 ± 8.47	112.48 ± 11.29	81.37 ± 32.49

**Table 4:** TC_50_ (nM) values of compounds **17a-b** against three cancer cell lines.

Compound	Cancer Cell Lines		
	HCT116	MDA-MB 231	H69AR
**17a**	40	<30	30
Arimetamycin A	250	320	90
**17b**	330	970	1010

## Data Availability

Not applicable.
